# Sex- and Age-Specific Differences in Mice Fed a Ketogenic Diet

**DOI:** 10.3390/nu16162731

**Published:** 2024-08-16

**Authors:** Kenyon W. Sprankle, Mya A. Knappenberger, Erica J. Locke, Jack H. Thompson, Madison F. Vinovrski, Kaylin Knapsack, Stephen C. Kolwicz

**Affiliations:** Heart and Muscle Metabolism Laboratory, Health Sciences Department, Ursinus College, Collegeville, PA 19426, USA; kenyonwsprankle@gmail.com (K.W.S.); mkk5658@psu.edu (M.A.K.); erlocke@ursinus.edu (E.J.L.); jathompson@ursinus.edu (J.H.T.); mavinovrski@ursinus.edu (M.F.V.); kaylinknapsack@gmail.com (K.K.)

**Keywords:** ketosis, glucose intolerance, lipid accumulation, ketone bodies, metabolism

## Abstract

The ketogenic diet (KD) is a high-fat, low-carbohydrate diet that results in the elevation of serum ketone bodies, known as ketosis. This metabolic consequence has been suggested as a method for treating neurological conditions, improving exercise performance, and facilitating weight loss for overweight individuals. However, since most research primarily uses male populations, little is known about the potential sex differences during the consumption of the KD. In addition, the effects of the KD on aging are relatively unexplored. Therefore, the purpose of this study was to explore sex- and age-specific differences in mice fed the KD. Male and female C57BL/6N mice at either 12 wks or 24 wks of age were randomly assigned to a KD (90% fat, 1% carbohydrate) or chow (13% fat, 60% carbohydrate) group for 6 wks. KD induced weight gain, increased adiposity, induced hyperlipidemia, caused lipid accumulation in the heart and liver, and led to glycogen depletion in the heart, liver, and muscle with varying degrees of changes depending on age and sex. While younger and older male mice on the KD were prone to glucose intolerance, the KD acutely improved rotarod performance in younger females. Overall, this study highlights potential sex and aging differences in the adaptation to the KD.

## 1. Introduction

The metabolism of ketone bodies has become an important subject of study in the scientific and medical community over the last decade. Studies suggest that the cellular usage of ketone bodies may be more energy efficient than fatty acids or glucose [1], which may be critical in the observations of improved recovery from ischemia [2], improvement in cardiac function in heart failure [3,4], and increased aerobic exercise performance [5]. These findings in the scientific literature, combined with information in the popular and mainstream media, are key drivers behind the rising popularity of diets and supplements that encourage ketone body metabolism. The suggested increase in metabolic efficiency of ketone bodies piques the interest of many individuals seeking to incorporate dietary strategies to increase weight loss, improve exercise capacity, and improve overall health.

Ketone bodies, namely acetone, acetoacetate (AcAC), and beta-hydroxybutyrate (β-OHB), are 4-carbon molecules which are synthesized through the process of ketogenesis in the liver and then broken down through the ketolysis pathway for energy production in peripheral tissues such as the heart and skeletal muscle [2]. β-OHB is the ketone body in the highest concentration in the blood and can be measured by several commercially available assay kits and by handheld ketone body measurement devices. While ketone body concentrations can be increased through fasting [6] and in response to acute exercise [7], nutritional strategies such as the ketogenic diet or ketone body supplements are also effective in inducing ketosis.

The ketogenic diet (KD) has been used in the past to treat a number of conditions in individuals, primarily that of epilepsy [8,9]. As a treatment for epilepsy, the diet has good efficacy for individuals in whom medication is ineffective [10]. In recent years, the diet has been used as a strategy for weight loss with some success [11]. The KD may be of potential benefit in obese individuals, but for individuals who are not suffering from obesity, negative consequences such as liver steatosis [11,12], issues with abnormal glucose homeostasis [11,12,13], and dyslipidemia [14,15] have been highlighted in research studies. Additionally, there are findings suggesting that the KD is not effective in improving aerobic [16,17,18] or anaerobic [19,20,21,22] performance in human subjects, underscoring the controversy of the diet.

In our previous study [6], we found that a short-term (i.e., 5 weeks) administration of the KD to 12–14-week-old female mice did not adversely affect exercise performance but resulted in several potential negative consequences such as increased adiposity, hypercholesterolemia, and increased cardiac and hepatic triglycerides. Since our previous study did not include a direct comparison to male mice, we expanded our work to support research examining sex as a biological variable [23,24]. Given some of the controversy regarding whether the KD improves longevity [25,26] or promotes cellular senescence [27], we included two commonly used age groups (i.e., 3 months and 6 months) in mouse studies to gain insight into the effects of KD in aging.

Despite the popularity of the KD in the mainstream media, studies assessing the consequences of the diet on overall health, activity, and behavior remain controversial. Moreover, since studies directly comparing the effects of the KD in male and female populations are scarce and the impact of the KD in aging is limited, this present study was designed to address some of the deficiencies in the current literature. Therefore, the purpose of the present study was to explore sex- and age-specific differences in physiology, metabolism, motor coordination, and behavior of mice fed the KD. Male and female mice at two different ages were fed a KD for 6 weeks. Anthropometric and metabolic assessments were made at the end of the study. In addition, the measures of motor coordination and activity using the rotarod performance test, and behavior using the open field test were conducted in both sexes and age cohorts. Overall, the findings reveal important sex and aging differences in mice fed the KD.

## 2. Materials and Methods

### 2.1. Study Design

Male (*n* = 52) and female (*n* = 51) C57BL/6-NCrl mice were used starting at 12 weeks or 24 weeks of age. The mice were randomly assigned to the control group which was fed a chow diet or a dietary intervention group which was fed a ketogenic diet for 6 weeks. To maximize rigor and reproducibility, the study was conducted a total of 4 times with different cohorts of animals included in each group. During the course of the study, all the mice were inspected daily by animal vivarium staff. All the mice were weighed on a weekly basis by the researchers and observed for signs of distress using the Body Conditioning Score. A cohort of mice had ketone bodies measured every two weeks and representative cohorts of mice were tested bi-weekly on the rotarod for a total duration of 6 weeks. After the 6-week study, open field testing and glucose tolerance tests were conducted on select cohorts of mice. The heart, liver, quadriceps muscle, and blood were harvested from all the mice. Biochemical assays were performed on the harvested tissues to determine triglyceride and glycogen content. The serum was tested for cholesterol, non-esterified fatty acids, triglycerides, insulin, and sex hormones. The tissue and serum analyses were performed in a blind fashion. All the mice randomly assigned to the chow or ketogenic diet groups completed the study and no adverse events occurred. All the animal experiments and procedures were approved prior to the start of the study by the Institutional Animal Care and Use Committee (IACUC) of Ursinus College.

### 2.2. Animal Model

The mice (*n* = 103) used for this study were either 12-week- or 24-week-old males and females obtained from an in-house C57BL/6-NCrl mouse colony. The mice were randomly assigned to either a chow-fed group (CD) or a ketogenic diet (KD) group. The male and female mice were separated into 4 groups: 12-week-old mice on chow diet (CD-12; male, *n* = 10; female, *n* = 9), 12-week-old mice on KD (KD-12; male, *n* = 11; female, *n* = 11), 24-week-old mice fed a chow diet (CD-24; male, *n* = 14, female, *n* = 13), and 24-week-old mice fed ketogenic diet (KD-24; male, *n* = 17; female, *n* = 18). The animals were housed 3–5 mice per cage and provided enrichment including nestlets and “mouse igloos” (Bio-Serve, Flemington, NJ, USA). All the mice were kept on a 12 h light/dark cycle and received food and water ab libitum.

### 2.3. Animal Diets

The KD was purchased from Research Diets (#D10070801, New Brunswick, NJ, USA) and comprised approximately 90% of the total calories from fats, <1% of total calories from carbohydrates, and 9% of total calories from protein. The fat source of the diet was derived from cocoa butter and yielded ~60% of the calories from saturated fatty acids. In comparison, the chow diet (Lab Diet, #5001) consisted of 13.6% calories from fat, 57.5% calories from carbohydrates, and 28.9% calories from protein. Additional macronutrient and micronutrient information for both diets appear in Table S1. Since the high-fat content of the KD is prone to oxidation, the food pellets for the KD groups were changed 3 times weekly. Food pellets for the chow groups were changed once weekly.

### 2.4. Measurement of Food and Caloric Intake

While most available KDs are in paste form, the diet used in this study was in a pellet form due to the high cocoa butter content, and allowed for an opportunity to monitor caloric intake. A measured quantity of food pellets from chow and KD was provided to each animal cage. After a two-day (for KD) or weekly period (for chow), the remaining pellets were collected and weighed. Food intake was determined by the difference in the mass of the pellets provided and the mass of the pellets remaining. The daily food intake per mouse was calculated by dividing the total food consumed by the number of mice in the cage. The caloric intake was calculated by multiplying the food intake by the manufacturer’s reported caloric yield: 3.36 kcal/g for chow and 6.7 kcal/g for KD.

### 2.5. Glucose Tolerance Testing

At the end of the 6-week feeding period, glucose tolerance tests (GTTs) were conducted in the mice following a 3 h fast. After measuring baseline blood glucose, the mice received an intraperitoneal injection of a 10% glucose solution at 10 mg/kg of body mass. After the injection, the blood glucose was measured via the tail tip method with a handheld glucometer at 15, 30, 60, 90, and 120 min [28]. Following the GTT, the mice were returned to the vivarium for a 24–48 h recovery period before any other procedures were conducted. Differences in glucose tolerance were assessed using an area under the curve (AUC) analysis.

### 2.6. Tissue Harvest

The mice were injected intraperitoneally with approximately 170 mg/kg of sodium phenobarbital. After confirming a negative response to the toe pinch test, the heart, liver, adipose tissue (i.e., epidydimal fat pad for males and inguinal fat pad for females), quadriceps muscle, and blood were harvested from all the mice. The masses of the heart, adipose tissue, and quadriceps were obtained and normalized to tibia length. All the tissues were frozen in liquid nitrogen and stored at −80 °C.

### 2.7. Rotarod Performance Test

Rotarod performance tests were performed bi-weekly for the duration of the 6-week period, in select cohorts of mice, using the ROTOR-ROD system (San Diego Instruments, San Diego, CA, USA). The rotarod increased in speed as the test continued. The mice had to balance and walk on the beam until they were unable to do so due to exhaustion or imbalance. The data for each mouse was displayed on the computer as the total time spent on the rotarod in seconds (i.e., latency to fall). The mice performed a total of three trials with a 5 min rest in between each trial. The reported data are an average of the three trials.

### 2.8. Open Field Test

The open field arena was a circular arena that consisted of a center zone and a border zone. The lights in the room were set to a pre-specified level before the testing began to allow for similar conditions across all the trials performed. The mice were placed into the central field and allowed to roam freely for 10 min. A video camera was fixed to the ceiling above the arena to track the movements of each mouse. An automated software (Ethovision XT 13.0, Noldus Technologies, Leesburg, VA, USA) was used to analyze the video recording. The analysis provided data regarding the time each mouse spent in the border versus center zone, the total distance each mouse covered during the 10 min period, and the average velocity.

### 2.9. Blood and Serum Analysis

Glucose and ketone bodies (beta-hydroxybutyrate) were measured in the blood obtained from the tail tip of all the mice using commercially available handheld meters (Contour Next glucometer for glucose and Keto-Mojo meter for ketone bodies). The blood harvested from the mice was incubated on ice for 15 min. Then, the blood was centrifuged for 10 min at 2000× *g* at 4 °C (Eppendorf Centrifuge, #5430R). After centrifugation, the serum was removed and stored at −80 °C until analysis. The mouse serum was assayed for non-esterified fatty acids (NEFAs), triglycerides, and total cholesterol using colorimetric kits purchased from a commercial vendor (Fujifilm Healthcare Americas Corporation, Lexington, MA, USA) Testosterone (#582701, Cayman Chemical, Ann Arbor, MI, USA) and estradiol (#ab108667, Abcam Inc., Waltham, MA, USA) concentrations were measured by commercially available ELISA-based kits.

### 2.10. Statistical Analysis

All the data are presented as the means ± standard error of the mean (SEM). The longitudinal data were analyzed via two-way repeated measures analysis of variance (ANOVA) followed by Tukey’s multiple comparison test. Data involving two independent variables (i.e., diet and age) were analyzed with a two-way ANOVA and Tukey’s post hoc test. Data involving three independent variables (i.e., diet, age, and sex) were analyzed with a three-way ANOVA and Tukey’s post hoc test. Statistical significance was tested at the *p* < 0.05 level. All the graphs were created, and analyses were performed using GraphPad Prism 10.2.

## 3. Results

### 3.1. Sex and Aging Differences in Anthropometric Measures in Response to Ketogenic Diet

**Figure 1:**
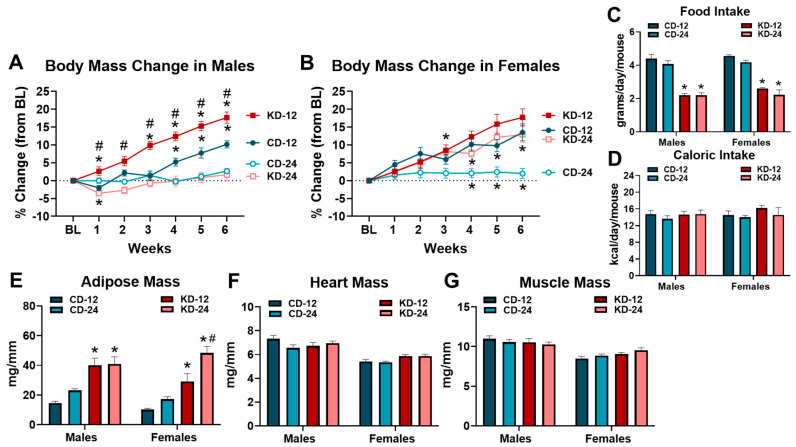
Changes in anthropometric measures due to ketogenic diet. (**A**) Weekly percent body mass change from baseline (BL) in male mice. (**B**) Weekly percent body mass change from BL in female mice. (**C**) Average daily food intake of chow and ketogenic diets in male and female mice. (**D**) Average daily caloric intake of chow and ketogenic diets in male and female mice. (**E**) Adipose tissue mass normalized to tibia length (TL) in mice. (**F**) Heart weight to TL ratios in mice. (**G**) Quadriceps to TL ratio in mice. CD-12, mice fed the chow diet starting at 12 weeks of age, *n* = 9–10. CD-24, mice fed the chow diet starting at 24 weeks of age, *n* = 13–14. KD-12, mice fed the ketogenic diet starting at 12 weeks of age, *n* = 11 in each group. KD-24, mice fed the ketogenic diet starting at 24 weeks of age, *n* = 17–18. * *p* < 0.05 vs. CD-12 or CD-24, # *p* < 0.05 vs. KD-12 or KD-24.

To assess the effects of the KD on the physical characteristics of male and female mice, body mass was assessed weekly, and the masses of the adipose tissue, heart, and quadriceps were obtained at the completion of the feeding period (Figure 1). Due to age and sex-related differences in body mass at baseline (Figure S1), body mass changes were analyzed as a percent change from the baseline (Figure 1A,B). Different patterns were noted in the male groups fed chow or KD. Male CD-12 and KD-12 groups increased body mass during the 6-week diet period, with the KD-12 group gaining significantly more weight than the CD-12 (Figure 1A). Neither CD-24 nor KD-24 increased weight significantly over the 6 weeks; however, the KD-24 group lost weight after the first week (Figure 1A). Interestingly, the body mass change was significantly greater in the KD-12 vs. KD-24 group (Figure 1A). Similar patterns of body mass changes were observed in the female mice, particularly for the chow-fed groups. CD-12 gained significantly more weight than CD-24; however, the body mass change was not significantly greater than the KD-12 group (Figure 1B). Unlike male mice, the body mass change in the KD-24 female group was significantly higher than CD-24 but was not significantly different than KD-12 (Figure 1B). Although food intake was lower in the KD groups due to the higher caloric content of the diet (Figure 1C), the estimated caloric intake was not statistically different among the groups (Figure 1D). The male and female KD-12 and KD-24 groups had significantly greater adipose tissue mass compared to chow-fed groups, while the female KD-24 group demonstrated the highest increase in adiposity (Figure 1E). The consumption of the KD did not significantly affect the heart or quadriceps mass in any group regardless of sex or age, although the heart and quadriceps masses were significantly lower in all the female groups compared to males (*p* < 0.05 main effect of sex; Figure 1F,G). In summary, the male mice fed a KD starting at 12 weeks of age experienced significant weight gain whereas the female mice of both ages experienced significant weight gain compared to the age-matched controls despite similar caloric intake. Although all the mice on a ketogenic diet have increased adiposity, the increase is greatest in 24-week-old females. Finally, 6 weeks of KD does not affect cardiac and skeletal muscle mass independent of age and sex. Overall, these data highlight sex and aging differences in body mass change and adiposity in response to the KD.

### 3.2. Changes in Serum Ketone Body and Lipid Metabolism after Ketogenic Diet

**Figure 2:**
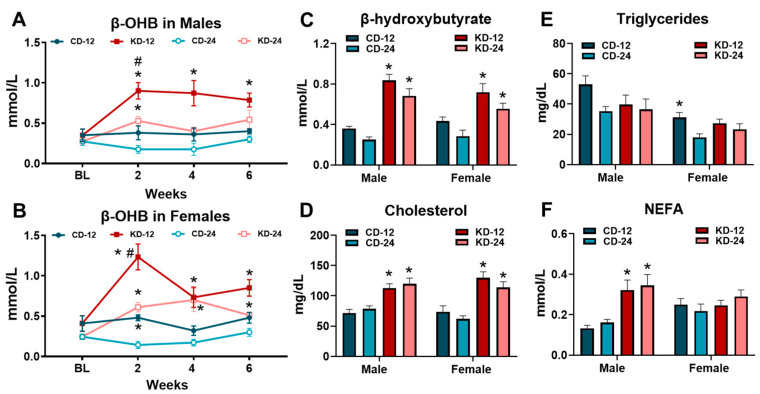
Ketogenic diet causes ketosis and hypercholesterolemia. β-hydroxybutyrate (β-OHB) was measured in blood in (**A**) male and (**B**) female mice at baseline (BL) and at 2-week intervals during the dietary feeding period. (**C**) β-hydroxybutyrate (β-OHB) measured in blood from male and female mice using a handheld meter prior to tissue harvest. (**D**) Cholesterol, (**E**) triglycerides, and (**F**) non-esterified fatty acids (NEFA) measured in the serum from blood collected at the time of tissue harvest. CD-12, mice fed the chow diet starting at 12 weeks of age, *n* = 5–10. CD-24, mice fed the chow diet starting at 24 weeks of age, *n* = 5–14. KD-12, mice fed the ketogenic diet starting at 12 weeks of age, *n* = 6–11. KD-24, mice fed the ketogenic diet starting at 24 weeks of age, *n* = 7–17. * *p* < 0.05 vs. CD-12 or CD-24, # *p* < 0.05 vs. KD-12.

To examine the time course of ketosis in response to the KD, we measured β-OHB at 2-week intervals over the feeding period in a cohort of animals. In the male and female mice, the KD led to significant increases in β-OHB after 2 weeks, with a more robust response in the younger KD-12 group (Figure 2A,B). At the end of the 6-week intervention, the male and female mice fed the KD had significantly higher β-OHB concentrations compared to their chow counterparts, with no significant sex and age differences (Figure 2C). The KD led to significant increases in serum cholesterol levels in the male and female mice of both age groups (Figure 2D). Serum triglycerides were generally unaffected by the KD in any group, despite the significant main effect of sex (Figure 2E). Non-esterified fatty acids (NEFAs) were significantly increased only in the male KD-12 and KD-24 groups (Figure 2F). In summary, these data show that the consumption of KD results in ketosis and hypercholesterolemia in both male and female mice, regardless of age. In addition, only male mice experience an increase in serum fatty acid levels.

### 3.3. Assessment of Glucose Metabolism in Mice Fed the Ketogenic Diet

**Figure 3:**
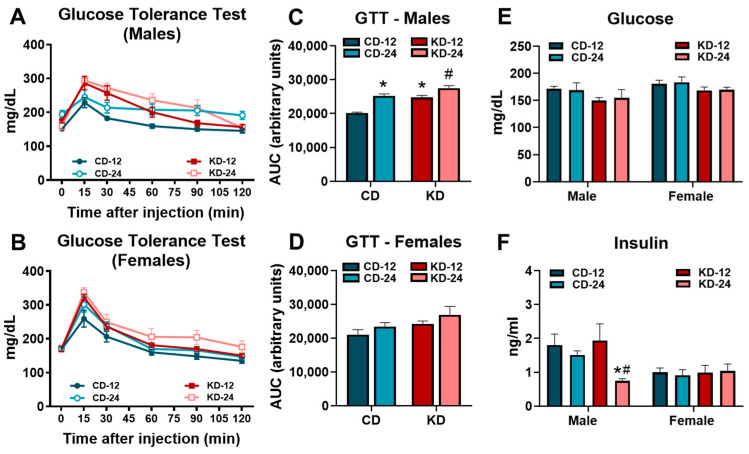
Glucose intolerance in male mice fed a ketogenic diet. (**A**) The glucose tolerance test performed in male mice. (**B**) The glucose tolerance test performed in female mice. (**C**) The area under the curve analysis of the glucose tolerance test in male mice. (**D**) The area under the curve analysis of the glucose tolerance test in female mice. (**E**) The blood glucose levels assessed by glucometer in mice prior to tissue harvest. (**F**) The serum insulin levels in mice from blood collected at the time of tissue harvest. CD-12, mice fed the chow diet starting at 12 weeks of age, *n* = 5–10. CD-24, mice fed the chow diet starting at 24 weeks of age, *n* = 6–10. KD-12, mice fed the ketogenic diet starting at 12 weeks of age, *n* = 7–10. KD-24, mice fed the ketogenic diet starting at 24 weeks of age, *n* = 7–10. * *p* < 0.05 vs. CD-12 or CD-24, # *p* < 0.05 vs. KD-12.

Based on the area under the curve (AUC) analysis, evidence of glucose intolerance was present in the CD-24 and KD-12 male mice compared to CD-12 with significantly greater glucose intolerance in the KD-24 versus KD-12 males (Figure 3A,C). In contrast, no significant differences were noted for the AUC analysis of the glucose tolerance test in female mice (Figure 3B,D). Despite the changes in glucose tolerance in males, glucose obtained prior to tissue harvest was not significantly different in the male and female mice fed the ketogenic diet regardless of age (Figure 3E). While serum insulin was significantly lower in the male KD-24 mice compared to the CD-24 and KD-12 cohorts, the insulin levels in females were unaffected by age or diet (Figure 3F). These findings show that male mice, but not female mice, are vulnerable to the development of glucose intolerance after 6 weeks of KD, which may be worse with aging.

### 3.4. Lipid Accumulation and Glycogen Depletion in Heart, Liver, and Skeletal Muscle

**Figure 4:**
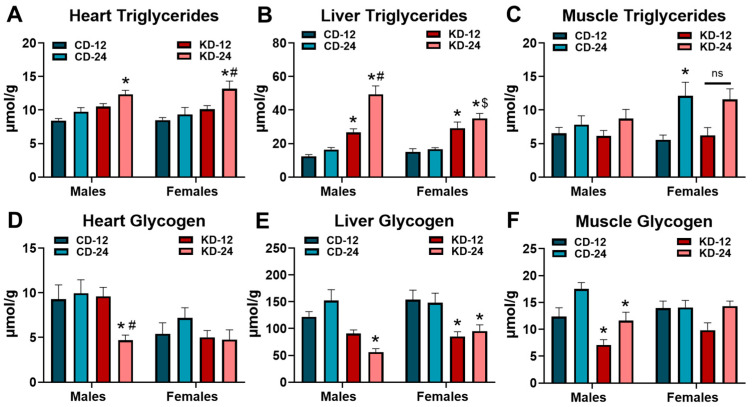
Lipid accumulation and glycogen depletion in the heart, liver, and muscle. Triglyceride content measured in extracts from the (**A**) heart; (**B**) liver; and (**C**) quadriceps muscle of male and female mice fed the ketogenic diet for 6 weeks. Glycogen content measured in extracts from the (**D**) heart; (**E**) liver; and (**F**) quadriceps muscle of male and female mice fed the ketogenic diet for 6 weeks. CD-12, mice fed the chow diet starting at 12 weeks of age, *n* = 9–11. CD-24, mice fed the chow diet starting at 24 weeks of age, *n* = 7–10. KD-12, mice fed the ketogenic diet starting at 12 weeks of age, *n* = 8–11. KD-24, mice fed the ketogenic diet starting at 24 weeks of age, *n* = 5–12. * *p* < 0.05 vs. CD-12 or CD-24 of same sex, # *p* < 0.05 vs. KD-12 of same sex. $ *p* < 0.05 vs. male KD-24. ns = *p* = 0.105.

Because of the higher fat and low carbohydrate content of the KD, critical metabolic organs could be susceptible to changes in endogenous storage. To this end, we assessed the triglyceride and glycogen content in the heart, liver, and skeletal muscle of the male and female KD groups (Figure 4). As shown in Figure 4A, heart triglycerides were significantly increased in the male and female KD-24 groups. While liver triglycerides were significantly higher in KD-fed groups regardless of age, liver triglycerides were elevated to a greater extent in male KD-24 compared to male KD-12 (Figure 4B). Interestingly, liver triglycerides in female KD-24 were not higher compared to female KD-12 but were significantly lower compared to male KD-24 (Figure 4B). The KD did not significantly alter triglycerides in the quadriceps muscle of the male and female mice with the caveat that the older female mice (i.e., CD-24 and KD-24) generally had higher muscle triglycerides (Figure 4C). The glycogen content in the hearts of male KD-24 were significantly lower than CD-24 and KD-12 counterparts (Figure 4D). The liver glycogen content was significantly lower in the male KD-24 and female KD-12 and KD-24 groups compared to the respective chow controls (Figure 4E). The glycogen content of the quadriceps muscle was significantly less in the male KD-12 and KD-24 groups but not in the female KD groups (Figure 4F). Taken together, these data highlight interesting changes in lipid accumulation in the heart and liver as well as glycogen depletion in the heart, liver, and skeletal muscle that are dependent upon the sex and age of the mice.

### 3.5. Behavioral Testing in Mice Fed the Ketogenic Diet

**Figure 5:**
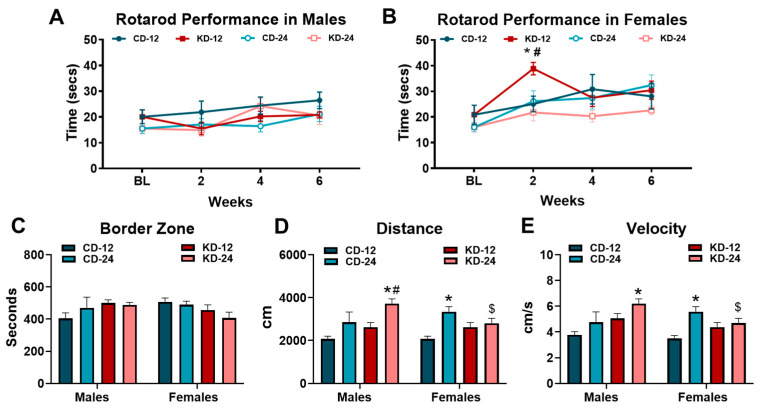
Behavioral analysis of mice on a ketogenic diet. (**A**) Rotarod performance in cohorts of male mice taken at baseline and every 2 weeks. (**B**) Rotarod performance in cohorts of female mice taken at baseline and every 2 weeks. (**C**) Average number of seconds spent in the border zone during open field testing. (**D**) Average distance traveled by mice measured during open field testing. (**E**) Average velocity of male and female cohorts during open field testing. CD-12, mice fed the chow diet starting at 12 weeks of age, *n* = 5 per group. CD-24, mice fed the chow diet starting at 24 weeks of age, *n* = 5–8. KD-12, mice fed the ketogenic diet starting at 12 weeks of age, *n* = 6–7. KD-24, mice fed the ketogenic diet starting at 24 weeks of age, *n* = 5–9. * *p* < 0.05 vs. CD-12 or CD-24 of same sex, # *p* < 0.05 vs. KD-12 of same sex. $ *p* < 0.05 vs. male KD-24.

To assess the effects of KD on motor coordination and activity, we subjected a cohort of mice of both sexes and ages to the rotarod performance and open field tests (Figure 5). Rotarod testing was performed at 2-week intervals over the 6-week feeding period while open field testing was conducted at the end of 6 weeks. As shown in Figure 5A, rotarod performance in the male mice fed the KD was similar to the chow groups despite a gradual improvement over the course of testing in all the groups (*p* < 0.05, main effect of time). Conversely, the response to KD in the female mice was more interesting. As shown in Figure 5B, the female KD-12 demonstrated a significant increase in rotarod performance at week 2 but declined to similar levels of the other female groups afterwards. The analysis of locomotor activity and behavior through the use of the open field test revealed differences in the male and female cohorts. The KD did not significantly alter the time spent in the border zone in male or female mice of both age groups (Figure 5C). However, male KD-24 mice accumulated higher total distances compared to male CD-24 and KD-12 mice which was also significantly greater than female KD-24. (Figure 5D). Similar patterns were observed with the velocity of movement during the open field testing with significant differences between the male and female KD-24 groups (Figure 5E). Findings from the rotarod performance test may suggest a possible acute effect of ketosis on performance in females while the results of the open field testing hint towards possible behavior changes as a result of both aging and the consumption of the KD in male mice.

### 3.6. Analysis of Sex Hormones after Ketogenic Diet

**Figure 6:**
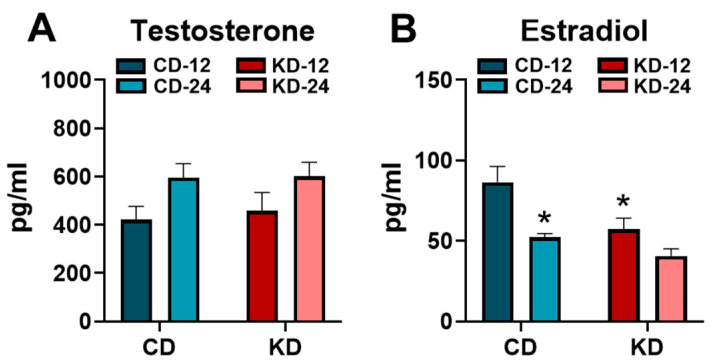
Serum testosterone and estradiol concentrations after the ketogenic diet. (**A**) The serum testosterone concentrations measured in male chow (CD)- or ketogenic diet (KD)-fed mice. (**B**) The serum estradiol concentrations measured in female chow (CD)- or ketogenic diet (KD)-fed mice. CD-12, mice fed the chow diet starting at 12 weeks of age, *n* = 7–9. CD-24, mice fed the chow diet starting at 24 weeks of age, *n* = 6–7. KD-12, mice fed the ketogenic diet starting at 12 weeks of age, *n* = 7–8. KD-24, mice fed the ketogenic diet starting at 24 weeks of age, *n* = 6–8. * *p* < 0.05 vs. CD-12.

Since a primary focus of the study was to assess sex differences, the concentrations of the sex hormones, testosterone and estradiol, were measured in a cohort of male and female mice, respectively (Figure 6). Serum testosterone was higher in the male CD-24 and KD-24 mice compared to the respective younger controls (*p* < 0.05, main effect of age) without any significant effects of the KD (Figure 6A). Conversely, serum estradiol was significantly lower in the female CD-24 and KD-12 groups compared to the female CD-12 (Figure 6B). All told, these data suggest that sex hormones are not affected by the KD in male mice but may lead to a lowering of sex hormones in younger female mice.

## 4. Discussion

The findings of this study reveal many impactful consequences of the KD over a short-term period. Unfortunately, the likelihood that the consumption of the KD will lead to potentially negative measures of health, which may be dependent upon sex and/or age, is possible based on the results of the present study. Specifically, our findings indicate that the short-term consumption of KD will lead to several undesirable changes including (1) increased adiposity; (2) hyperlipidemia; (3) lipid accumulation in metabolic organs; (4) glycogen depletion in metabolic organs; (5) glucose intolerance; (6) altered activity and behavior; and (7) altered sex hormones. The degree of these potentially deleterious effects is dependent upon the sex and age of the animal. In total, our results identify numerous negative consequences of short-term consumption of KD and highlight important sex and aging effects that require further study.

The effect of KDs on weight loss and obesity remains an interesting point of debate. While studies in humans tend to report that KDs contribute to weight loss in various populations [29,30], studies in mice present divergent results. In our current study, the KD progressively increased body mass in younger male and in both younger and older female mice, which was associated with increased adipose tissue mass. These findings are consistent with our previous work in younger female mice [6] as well as other reports in male mice [31,32]. However, other work shows reduced body weight and adipose mass after KD in males but not females [33]. Interestingly, this study used a KD with 3% of the calories from carbohydrates with similar fat content [33], suggesting that the carbohydrate content of the KD could be a potential factor. In support of this, human studies, which typically are more consistent with low-carbohydrate diets rather than true ketogenic diets, show beneficial effects on weight loss with reduced rather than very low carbohydrate intakes [29]. However, several mouse studies demonstrate that body mass and adiposity in KDs with a carbohydrate content of less than 1% of the total calories is similar to chow-fed controls but significantly less than high-fat diets containing high sucrose [12,34,35], which suggests that factors beyond carbohydrate content is involved. Fatty acid composition, including the relevant amounts of saturated, monounsaturated, and polyunsaturated fatty acids, or the source (i.e., medium versus long chain fatty acids) could also be important. Another potential confounding variable in the studies could be the mouse strain. We purposefully selected the C57BL/6N strain over the popular C57BL/6J to avoid the potential confounding variable of the mutations in the nicotinamide nucleotide transhydrogenase (Nnt) protein, since these two strains have different cardiac responses during pressure-overload hypertrophy [36], which may be related to protection from oxidative stress [37]. Therefore, the consideration of mouse strain may be an additional variable to consider when interpreting animal studies. Of note, we observed very minor changes in body mass over the 6 weeks in the older chow-fed groups as well as the older male KD group. Based on this, the age of the mouse at the initiation of any dietary intervention could be another important consideration in animal research.

Our analyses of systemic cellular metabolism using various markers in blood and serum yielded results that should present a pause in consideration of the use of KD. As we [6] and others have reported [14,15,38], the consumption of KD induced hypercholesterolemia in both male and female mice. Moreover, male mice, regardless of age, experience an increase in serum fatty acid levels. This evidence of hyperlipidemia, particularly in males, could offer an increased risk of the development of chronic disease, including coronary artery disease [39]. Another significant observation in our study is the development of glucose intolerance in male mice after just 6 weeks of the KD, a finding that was worsened with age. Our observation of reduced insulin levels in the older male mice combined with glucose intolerance could be an indication of the development of type 2 diabetes [13]. In support of this possibility, the findings of abnormal glucose metabolism in male mice fed a KD have been previously reported [12,15,31,32]. Interestingly, female mice, despite increased body weight and adiposity, were protected against glucose intolerance.

The high fat combined with the low carbohydrate content of the KD potentially predisposes critical metabolic tissues, namely the heart, liver, and skeletal muscle, with an over/under supply of important metabolic substrates. As such, lipid accumulation and the possible development of “lipotoxicity” could manifest. Glycogen depletion in the heart and skeletal muscle could present a challenge to increased metabolic demand, while glycogen depletion in the liver could affect glucose homeostasis. Our findings are consistent with other reports [12,31,32] demonstrating lipid accumulation in the liver regardless of sex or age. In contrast, the age of the mice seems to be a more important factor in the elevation of cardiac triglycerides, especially in female mice. While decreased liver glycogen content with the KD is generally consistent across both sexes and age groups, cardiac glycogen is relatively unaffected except for older male mice. Interestingly, the accumulation of triglycerides in skeletal muscle is minor, while muscle glycogen depletion seems to be dependent upon sex. Overall, the findings suggest that changes in the endogenous storage of triglycerides and glycogen in response to KD are more pronounced in older mice, with the greatest effect observed in older males. How these changes contribute to cellular function or the development of chronic diseases is not clear. However, the concerns about high cardiac lipids and reduced glycogen levels could be a factor in the recovery from cardiac ischemia [2].

While our present work demonstrated no improvement in exercise performance in female mice fed a KD [6], other studies have suggested a positive effect on exercise [40,41] using treadmill protocols. Although the effects of KD on aerobic exercise performance may be inconsistent, KD may promote other aspects of physical activity. Therefore, we elected to employ the rotarod performance and open field tests, two commonly used procedures in rodent research to examine motor performance, locomotor activity, and behavior [42,43]. Overall, we did not observe a significant effect of the KD on rotarod performance in the male or female mice, which contrasts previous reports [44,45]. However, we did see a transient increase in rotarod performance in younger KD-fed females. Interestingly, this improvement in performance was associated with the maximum peak of blood β-OHB concentration (see Figure 2B), which may suggest that performance enhancements are dependent upon the level of ketone body concentrations. In support of this, previous studies demonstrating improvement in measures of performance had subjects with ketone body concentrations in the 3–4 mM range [5,46,47], which is much greater than what was achieved by feeding the KD to the mice in our study. The increase in adipose tissue mass observed in our animals could be easily explained by decreased activity. Therefore, we used the open field test to examine potential changes in spontaneous locomotor activity due to the consumption of the KD. Based on the results of the open field test, the increase in adiposity is not easily explained by decreased locomotor activity, determined by the total distance achieved and velocity of movement. However, locomotor activity was affected by age, diet, and sex. The older, chow-fed female mice covered more distance and at a faster velocity. Interestingly, the KD appeared to have the greatest effect on the older, male mice. Since KD has been suggested to improve mental health, including depression and anxiety [48], we analyzed the time in the border zone, typically used as an indicator of anxiety during open field testing [49]. However, we did not observe any significant effects due to the KD, sex, or age. While our findings from the open field test are difficult to interpret, most studies do not find a significant effect of KD in various parameters measured with open field testing [50]. The inclusion of additional behavioral testing and more careful evaluation of the measured parameters could help in the overall interpretation.

In our study, we included the assessment of sex hormones in order to improve the rigor associated with the analysis of sex as a biological variable. While we did not attempt to control for sex hormones in the study, we did perform assessments on mice at approximately the same age and at the same time of day. Overall, we did not observe great variation in the concentrations of sex hormones. Interestingly, our data seem to suggest a potential effect of KD on sex hormones in females, but not male mice. Serum testosterone was elevated in both the older chow and KD groups, suggesting a possible aging effect. A previous study assessed serum testosterone in mice and found that levels were highest at 5 months of age with similar levels between the 1.5- and 12-month-old mice [51]. At the time of analysis, our mice were approximately 4.5 vs. 7.5 months of age, so whether age is a possible explanation for the findings is not clear, since there are limited data comparing testosterone levels between 6 months and 12 months of age. In our study, estradiol levels were decreased with age, which is consistent with previous work that shows a reduction after 5 months of age [52]. Surprisingly, the KD led to reduced estradiol levels in younger females. While the reason for this is not clear, we did not carefully monitor the estrus cycle in the study, so we cannot exclude this as a contributing variable.

A major point of discussion that may affect the overall interpretations of our study revolves around the selected diets. We elected to use a standard rodent chow diet for the comparison group rather than a purified control diet which is often recommended [53]. We intentionally chose this diet to more closely mimic a dietary change from a “normal diet” to a KD. While control purified diets are useful in matching ingredients and micronutrient composition, many “in-stock” diets available from commercial vendors include a higher carbohydrate content, consisting mostly of sucrose, and lower fat content which could represent a significant dietary change from the typical chow diets. Consistent with this, a previous report found that a purified control diet caused glucose intolerance and increased serum lipid levels in mice [54]. Therefore, the choice of a control diet could be critical in evaluating metabolic responses to dietary interventions. To alleviate these concerns, researchers can often customize control diets in order to match the macronutrient content and ingredients of the KD or high-fat diet. Overall, this highlights the need for more careful attention to the nutritional profile of both the control and intervention diets used in animal studies. In addition, researchers should consider including both control groups (i.e., chow and control diet) or performing preliminary studies assessing the potential effects of purified control diets.

There are several additional potential limitations of our study. First, the chow and KD differ in other dietary aspects that are worth noting. The chow and KD do differ in protein content, although the lower protein content in the KD is not considered to be protein deficient. Protein content and other micronutrients, such as choline, are known to influence the effects of KD [55]. The source of protein in the KD is casein, while a variety of sources, primarily soy, is used in the chow diet. This is of interest since casein may attenuate weight gain and adiposity [56] but promote hyperlipidemia and lipid accumulation in comparison to soy protein [57] in mouse diet-induced obesity studies. The fiber source of the diets utilized in our study also differs. In the KD, the insoluble fiber, cellulose, is the primary source whereas the chow diet contains various sources of both soluble and insoluble fiber. While the fiber content of our KD is not reduced compared to the chow, the fiber source differences could confound the interpretation of metabolic data in dietary feeding studies [58]. Given the importance of nutritional profile on the microbiome [59], we also cannot exclude the influence of changes in the gut microbiota on the outcomes [31]. Certainly, additional research in this area is needed. Second, although we attempted to assess food consumption and caloric intake in the mice, the accurate assessment of food intake or calories consumed without metabolic cages is difficult since some mice exhibit the abnormal behavior known as “food grinding” [60], which may lead to an overproduction of food waste and overestimate food and caloric intake. Third, we only utilized 6 weeks of KD in our study, which is consistent with the reported “keto-adaptation” time [29]. The possibility exists that longer dietary feeding times could alter the results. Last, we only examined two ages of mice (i.e., 3 months and 6 months), which are still relatively early in the lifespan. The additional examination of mice at a later age or with other chronic conditions could be informative.

## 5. Conclusions

The present study reports several negative consequences of KD in both male and female mice, which could be worsened with age. The KD increases adiposity, induces hypercholesterolemia, lipid accumulation in the heart and liver, and hepatic glycogen depletion regardless of sex with some of the changes more pronounced with increasing age. Male mice fed a KD are particularly vulnerable to the development of glucose intolerance, particularly older mice that also experience a decrease in serum insulin. While older female mice are prone to increased adiposity and increased cardiac lipid accumulation on the KD, they are protected against the development of glucose intolerance and hepatic lipid accumulation. In total, male mice, particularly older male mice, appear to be most susceptible to the negative consequences of KD.

## Data Availability

The original contributions presented in the study are included in the article/Supplementary Materials, further inquiries can be directed to the corresponding author.

## Supplementary Materials

The following supporting information can be downloaded at: https://www.mdpi.com/article/10.3390/nu16162731/s1, Table S1: Macronutrient and Micronutrient Profile of Chow and Ketogenic Diet; Figure S1: Body Masses of Male and Female Mice.
