# Supplementary material for: Sex- and Age-Specific Differences in Mice Fed a Ketogenic Diet
Source: Nutrients. 2024 Aug 16;16(16):2731. doi: 10.3390/nu16162731 (PMC11357043; doi:10.3390/nu16162731)
Supplement: Supplementary file 1 [file nutrients-16-02731-s001.zip › nutrients-3141686-supplementary.pdf]

Table S1. Macronutrient and micronutrient profile of the Chow and Ketogenic Diet

Macronutrients	Chow Diet (CD)	Ketogenic Diet (KD)
**Protein, g/100g**	24.10	16.80
**Saturated fat, g/100g**	1.39	37.70
**Monounsaturated fat, g/100g**	1.52	20.10
**Polyunsaturated fat, g/100g**	1.39	4.20
**Omega 3 fatty acid, g/100g**	0.31	0.40
**Cholesterol, mg/kg**	196	0.00
**Sucrose, g/100g**	3.25	0.79
**Starch, g/100g**	21.90	0.00
**Fiber, g/100g**	5.30	8.26
**Micronutrients**	**Chow Diet (CD)**	**Ketogenic Diet (KD)**
**Calcium, g/100g**	0.95	1.00
**Phosphorous, g/100g**	0.67	0.49
**Potassium, g/100g**	1.22	0.99
**Magnesium, g/100g**	0.21	0.08
**Sulfur, g/100g**	0.33	0.05
**Sodium, g/100g**	0.39	0.17
**Chloride, g/100g**	0.65	0.26
**Iron, mg/kg**	240	61
**Iodine, mg/kg**	1.00	0.33
**Zinc, mg/kg**	75	48
**Vitamin A, IU/g**	18.0	6.6
**Vitamin D, IU/g**	4.60	1.65
**Vitamin E, IU/kg**	42.00	82.57
**Thiamin, mg/kg**	16	9.9
**Riboflavin, mg/kg**	4.7	9.9
**Niacin, mg/kg**	130	50
**Pantothenic Acid, mg/kg**	24	26.4
**Choline, mg/kg**	1840	1350
**Folic Acid, mg/kg**	7.2	3.3
**Pyridoxine, mg/kg**	6.1	11.6
**Biotin, mg/kg**	0.30	0.33
**Cyanocobalamin, mcg/kg**	51	16.5

Macronutrient and micronutrient profiles were calculated based on online datasheets provided by each manufacturer (i.e., Lab Diet for #5001 chow diet (CD), and Research Diets for #D10070801 ketogenic diet (KD). Published values were converted to grams per 100 grams (g/100g), milligrams per kilograms (mg/kg), or International Units per gram or kilogram (IU/g, IU/kg) for comparison. Both manufacturers report that the published values are estimates based on periodic chemistry analysis and the ingredient lists. The vitamin and mineral contents of the KD is calculated based on the added supplement mixes (#V10001C and S10026) and does not include trace amounts of vitamins and minerals from other ingredients of the diet.

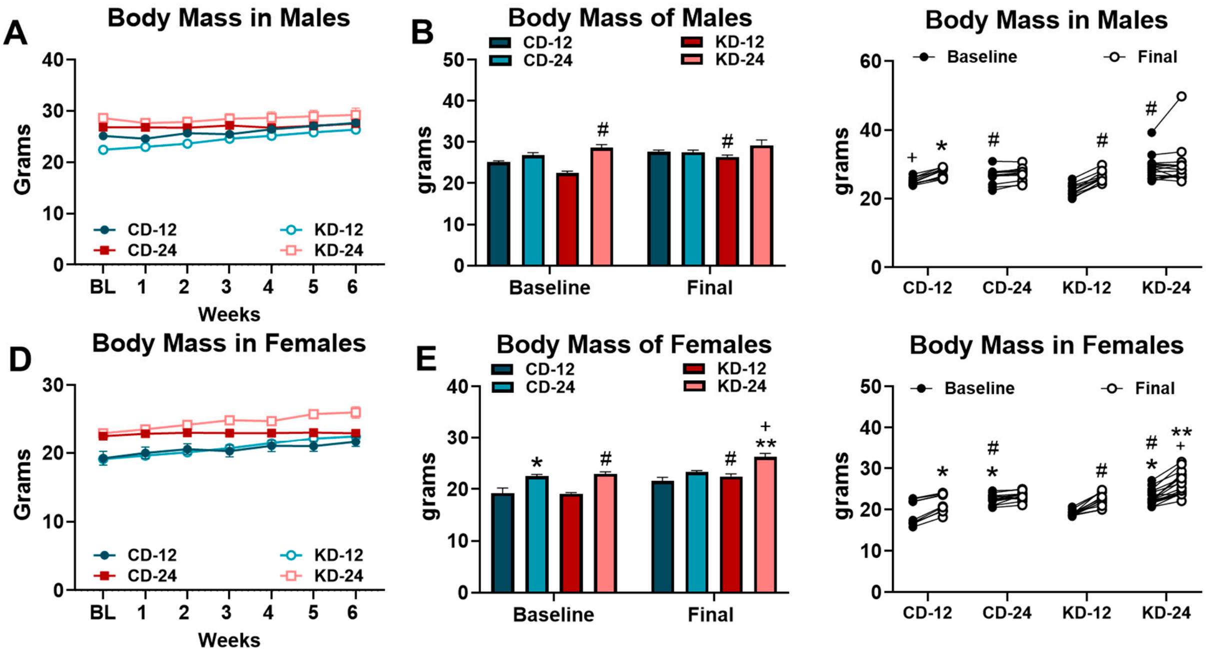

Figure S1. Baseline and Final Body Mass of Male and Female Mice. A) Weekly body mass of male mice over the 6 week study. B) Body mass of males measured at baseline and at the end of 6 weeks (Final). Younger male mice fed the ketogenic diet (KD-12) experienced a significant increase in body mass at the end of 6 weeks, while the other groups did not. C) Comparison of individual baseline and final body masses for male mice. D) Weekly body mass of female mice over the 6 week study. E) Body mass of females measured at baseline and at the end of 6 weeks (Final). As expected, older female mice (CD-24 and KD-24) had significantly higher body masses at baseline. Both female ketogenic diet groups (KD-12 and KD-24) experienced a significant increase in body mass after 6 weeks. F) Comparison of individual baseline and final body masses for female mice. KD-24 females were significantly heavier than KD-12, likely, in part, due to age differences. CD-12, mice fed the chow diet starting at 12 weeks of age, n= 9-10. CD-24, mice fed the chow diet starting at 24 weeks of age, n= 13-14. KD-12, mice fed the ketogenic diet starting at 12 weeks of age, n= 11 each group. KD-24, mice fed the ketogenic diet starting at 24 weeks of age, n= 17-18. * $P < 0.05$ vs. CD-12 at baseline, # $P < 0.05$ vs. KD-12 at baseline, + $P < 0.05$ vs. KD-24 at baseline, ** $P < 0.05$ vs. KD-12 and CD-24 at final.
